# Supplementary material for: Evaluation of a Proportional–Integral–Derivative Controller for Hemorrhage Resuscitation Using a Hardware-in-Loop Test Platform
Source: J Pers Med. 2022 Jun 16;12(6):979. doi: 10.3390/jpm12060979 (PMC9224865; doi:10.3390/jpm12060979)
Supplement: Supplementary file 1 [file jpm-12-00979-s001.zip › jpm-1768670-supplementary.pdf]

Supplementary Table S1. Average performance metrics for two PID configurations for test scenario 1

Scenario 1		
	Conservative	Aggressive
MDPE (%)	0.16	1.83
MDAPE (%)	0.58	2.04
Steady-state overshoot (%)	1.28	2.01
Steady-state undershoot (%)	-31.23	-32.07
Target overshoot (%)	1.07	3.50
Effectiveness (%)	92.85	96.20
Wobble (%)	0.35	0.56
End-state divergence (%/hr)	-0.41	0.51
Rise time efficiency (min)	3.17	2.22
Volume efficiency	3.05	3.11
Median infusion rate (mL/min)	4.06	0.00
Mean infusion rate (mL/min)	17.50	18.45
Area above target pressure (min)	0.17	0.98
Area below target pressure (min)	-1.18	-0.67
Area to rise time (min)	-0.62	-0.41
Infusion rate variability (%)	5.40	15.78

Supplementary Table S2. Average performance metrics for two PID configurations for test scenario 2

Scenario 2		
	Conservative	Aggressive
MDPE (%)	-2.71	-0.44
MDAPE (%)	2.71	1.21
Steady-state overshoot (%)	1.85	1.59
Steady-state undershoot (%)	-29.51	-30.81
Target overshoot (%)	0.25	1.15
Effectiveness (%)	79.07	88.61
Wobble (%)	0.75	0.63
End-state divergence (%/hr)	-4.40	2.46

**Rise time efficiency (min)**	3.56	2.44
**Volume efficiency**	33.66	41.31
**Median infusion rate (mL/min)**	27.17	32.99
**Mean infusion rate (mL/min)**	45.73	56.37
**Area above target pressure (min)**	0.01	0.09
**Area below target pressure (min)**	-1.57	-0.90
**Area to rise time (min)**	-0.79	-0.51
**Infusion rate variability (%)**	6.95	33.28

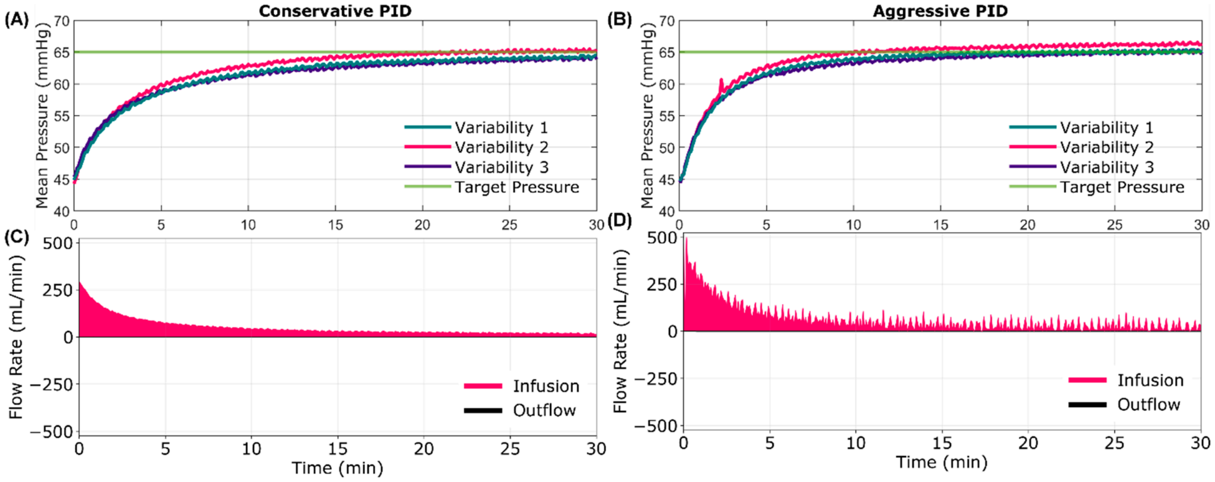

Supplementary Figure S1. Results for Scenario 2. Results for the Conservative (A, C) and Aggressive (B, D) tuned PID are shown for (A,B) MAP and (C,D) infusion and outflow rate as the controller tries to reach a target MAP of 65mmHg. For MAP plots, each of three subject variability runs are plotted individually while a single representative run is shown for the inflow/outflow plots.

Supplementary Table S3. Average performance metrics for two PID configurations for test scenario 3

Scenario 3		
	**Conservative**	**Aggressive**
**MDPE (%)**	-1.96	-0.54
**MDAPE (%)**	1.99	0.85
**Steady-state overshoot (%)**	3.02	2.10
**Steady-state undershoot (%)**	-2.01	-2.07
**Target overshoot (%)**	0.96	1.51

Effectiveness (%)	100.28	100.28
Wobble (%)	0.85	0.72
End-state divergence (%/hr)	-4.72	4.18
Rise time efficiency (min)	NA	NA
Volume efficiency	0.93	1.04
Median infusion rate (mL/min)	17.04	8.67
Mean infusion rate (mL/min)	17.23	19.65
Area above target pressure (min)	0.00	0.06
Area below target pressure (min)	-0.61	-0.23
Area to rise time (min)	0.00	0.00
Infusion rate variability (%)	2.94	22.61

Supplementary Table S4. Average performance metrics for two PID configurations for test scenario 4

Scenario 4		
	Conservative	Aggressive
MDPE (%)	-1.97	0.02
MDAPE (%)	1.98	0.93
Steady-state overshoot (%)	2.26	2.00
Steady-state undershoot (%)	-30.34	-31.19
Target overshoot (%)	0.92	2.20
Effectiveness (%)	82.13	90.74
Wobble (%)	0.79	0.70
End-state divergence (%/hr)	0.65	7.55
Rise time efficiency (min)	3.56	2.31
Volume efficiency	2.53	2.54
Median infusion rate (mL/min)	20.97	15.52
Mean infusion rate (mL/min)	39.69	42.60
Area above target pressure (min)	0.01	0.14
Area below target pressure (min)	-1.36	-0.69
Area to rise time (min)	-0.76	-0.45

**Infusion rate variability (%)**	7.17	29.59

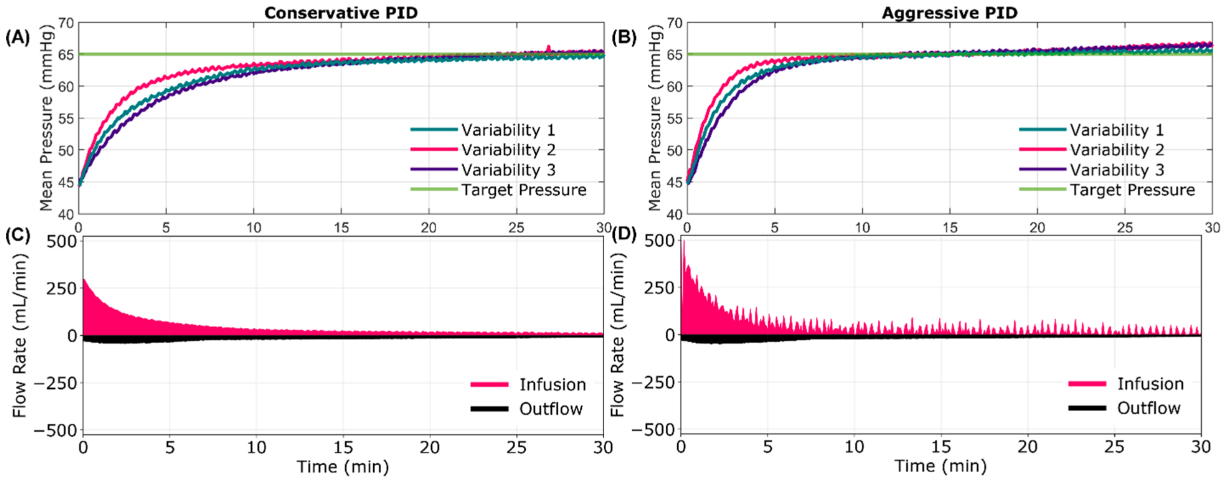

Supplementary Figure S2. Results for Scenario 4. Results for the Conservative (A, C) and Aggressive (B, D) tuned PID are shown for (A,B) MAP and (C,D) infusion and outflow rate as the controller tries to reach a target MAP of 65mmHg. For MAP plots, each of three subject variability runs are plotted individually while a single representative run is shown for the inflow/outflow plots.

Supplementary Table S5. Average performance metrics for two PID configurations for test scenario 5

Scenario 5		
	**Conservative**	**Aggressive**
**MDPE (%)**	-4.06	-1.27
**MDAPE (%)**	4.06	1.50
**Steady-state overshoot (%)**	2.22	1.82
**Steady-state undershoot (%)**	-28.80	-30.06
**Target overshoot (%)**	0.15	0.81
**Effectiveness (%)**	73.70	87.04
**Wobble (%)**	0.94	0.72
**End-state divergence (%/hr)**	-7.28	0.04
**Rise time efficiency (min)**	3.83	2.67
**Volume efficiency**	3.61	4.10
**Median infusion rate (mL/min)**	38.19	45.48
**Mean infusion rate (mL/min)**	55.02	66.90

**Area above target pressure (min)**	0.00	0.04
**Area below target pressure (min)**	-1.89	-1.05
**Area to rise time (min)**	-0.93	-0.54
**Infusion rate variability (%)**	6.67	34.56

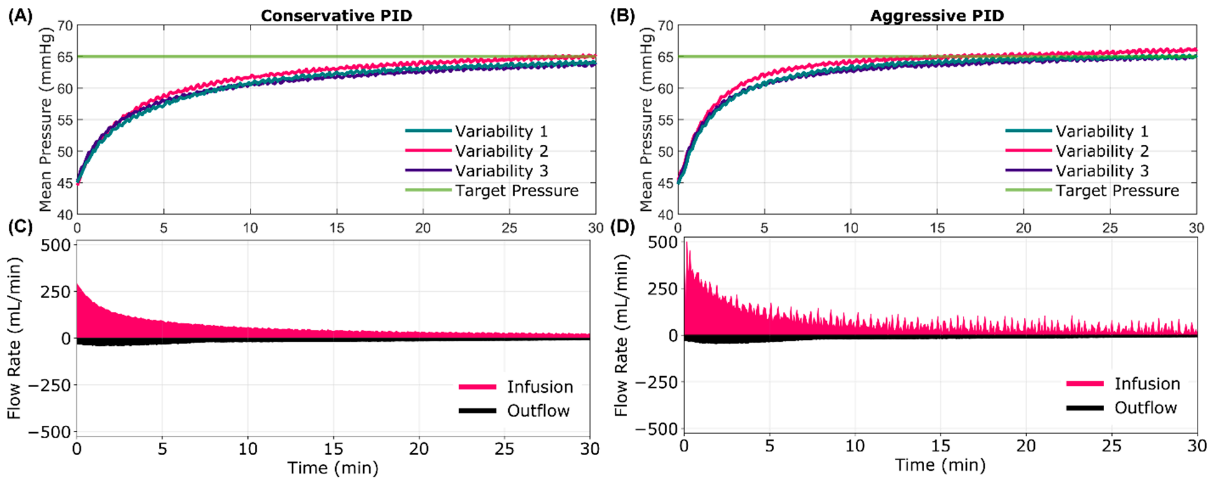

Supplementary Figure S3. Results for Scenario 5. Results for the Conservative (A, C) and Aggressive (B, D) tuned PID are shown for (A,B) MAP and (C,D) infusion and outflow rate as the controller tries to reach a target MAP of 65mmHg. For MAP plots, each of three subject variability runs are plotted individually while a single representative run is shown for the inflow/outflow plots.

Supplementary Table S6. Average performance metrics for two PID configurations for test scenario 6

Scenario 6		
	**Conservative**	**Aggressive**
**MDPE (%)**	-3.74	-1.51
**MDAPE (%)**	3.74	1.57
**Steady-state overshoot (%)**	4.56	2.95
**Steady-state undershoot (%)**	-3.75	-3.39
**Target overshoot (%)**	0.53	1.28
**Effectiveness (%)**	94.72	100.28
**Wobble (%)**	1.62	1.13
**End-state divergence (%/hr)**	-6.64	8.22
**Rise time efficiency (min)**	NA	NA

**Volume efficiency**	0.96	1.00
**Median infusion rate (mL/min)**	32.39	30.41
**Mean infusion rate (mL/min)**	32.58	35.48
**Area above target pressure (min)**	0.00	0.04
**Area below target pressure (min)**	-1.14	-0.52
**Area to rise time (min)**	0.00	0.00
**Infusion rate variability (%)**	3.68	26.02

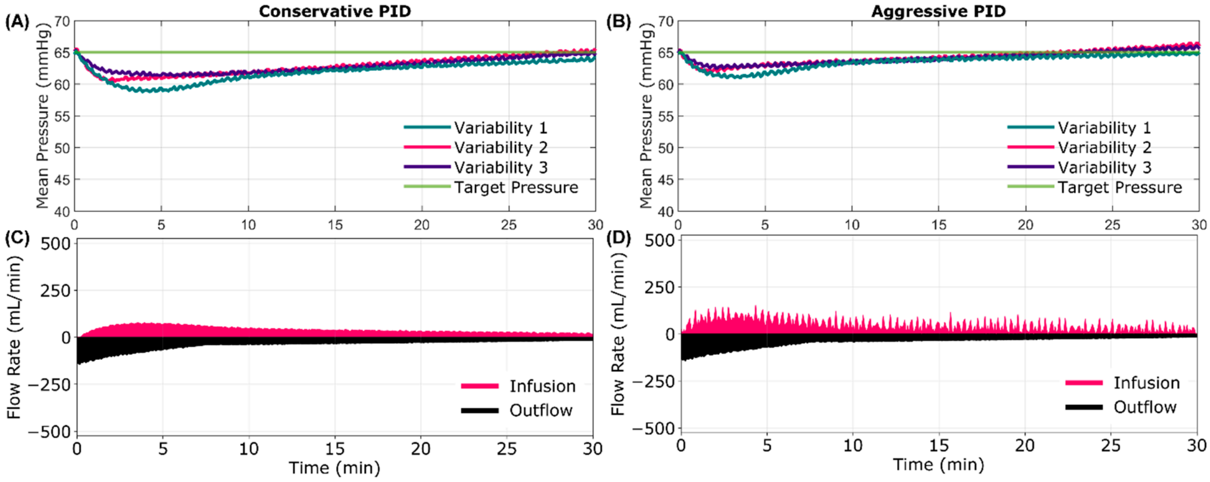

Supplementary Figure S4. Results for Scenario 6. Results for the Conservative (A, C) and Aggressive (B, D) tuned PID are shown for (A,B) MAP and (C,D) infusion and outflow rate as the controller tries to reach a target MAP of 65mmHg. For MAP plots, each of three subject variability runs are plotted individually while a single representative run is shown for the inflow/outflow plots.

Supplementary Table S7. Average performance metrics for two PID configurations for test scenario 7

**Scenario 7**		
	**Conservative**	**Aggressive**
**MDPE (%)**	-3.76	-1.01
**MDAPE (%)**	3.76	1.38
**Steady-state overshoot (%)**	2.46	2.46
**Steady-state undershoot (%)**	-29.41	-30.47
**Target overshoot (%)**	0.54	1.97
**Effectiveness (%)**	75.65	88.89
**Wobble (%)**	1.13	0.88

**End-state divergence (%/hr)**	1.12	10.56
**Rise time efficiency (min)**	4.22	2.58
**Volume efficiency**	1.80	1.79
**Median infusion rate (mL/min)**	35.71	35.47
**Mean infusion rate (mL/min)**	52.39	57.00
**Area above target pressure (min)**	0.01	0.07
**Area below target pressure (min)**	-1.80	-0.91
**Area to rise time (min)**	-0.96	-0.51
**Infusion rate variability (%)**	6.81	32.51

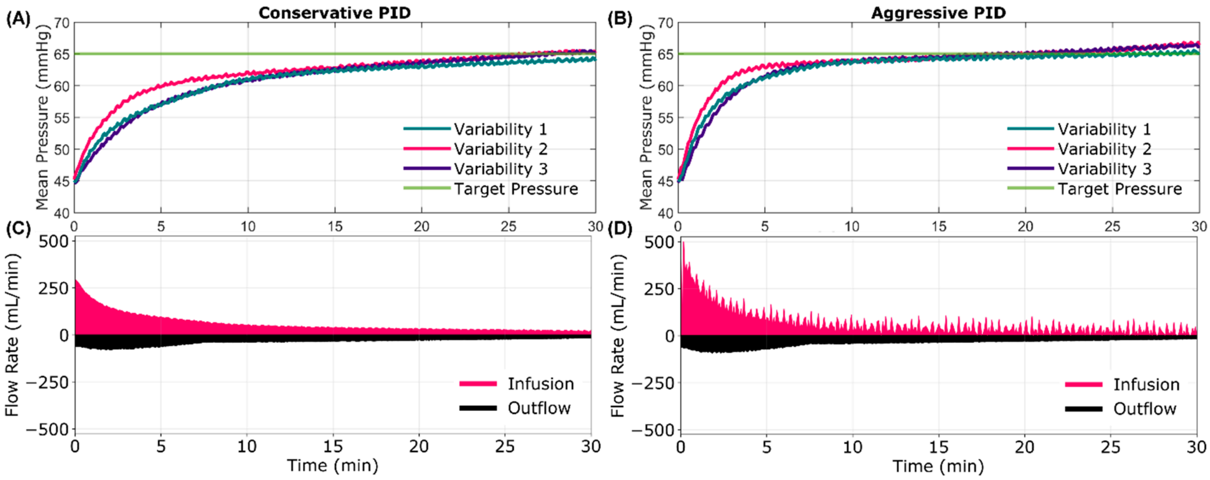

Supplementary Figure S5. Results for Scenario 7. Results for the Conservative (A, C) and Aggressive (B, D) tuned PID are shown for (A,B) MAP and (C,D) infusion and outflow rate as the controller tries to reach a target MAP of 65mmHg. For MAP plots, each of three subject variability runs are plotted individually while a single representative run is shown for the inflow/outflow plots.

Supplementary Table S8. Average performance metrics for two PID configurations for test scenario 8

Scenario 8		
	**Conservative**	**Aggressive**
**MDPE (%)**	-5.47	-1.84
**MDAPE (%)**	5.47	1.90
**Steady-state overshoot (%)**	2.47	2.07
**Steady-state undershoot (%)**	-28.29	-30.15

Target overshoot (%)	0.04	0.71
Effectiveness (%)	66.30	84.63
Wobble (%)	1.11	0.90
End-state divergence (%/hr)	-12.03	-0.44
Rise time efficiency (min)	4.36	3.00
Volume efficiency	2.32	2.54
Median infusion rate (mL/min)	49.63	57.49
Mean infusion rate (mL/min)	65.63	78.04
Area above target pressure (min)	0.00	0.02
Area below target pressure (min)	-2.27	-1.23
Area to rise time (min)	-1.19	-0.62
Infusion rate variability (%)	6.53	35.04

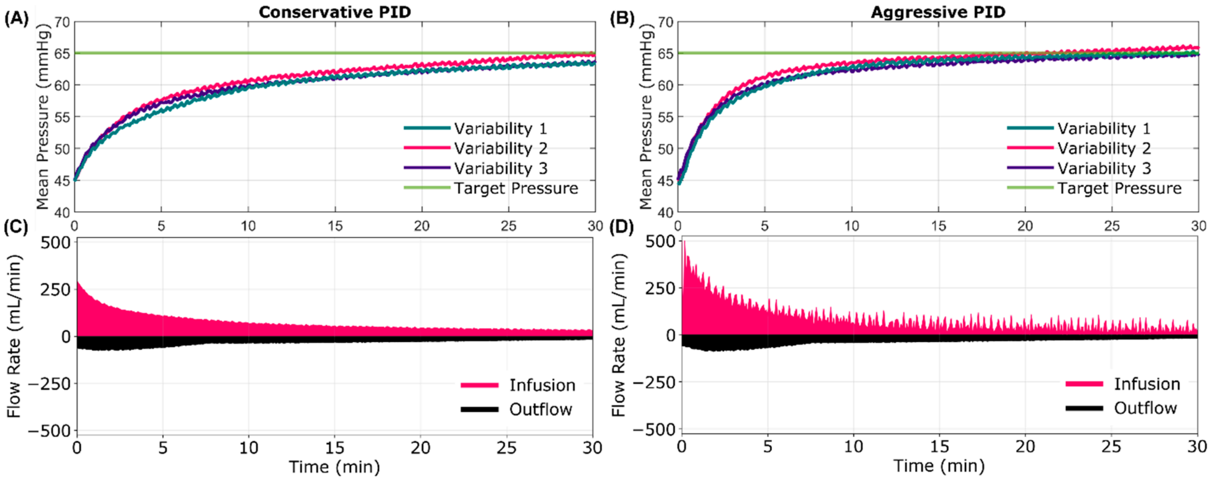

Supplementary Figure S6. Results for Scenario 8. Results for the Conservative (A, C) and Aggressive (B, D) tuned PID are shown for (A,B) MAP and (C,D) infusion and outflow rate as the controller tries to reach a target MAP of 65mmHg. For MAP plots, each of three subject variability runs are plotted individually while a single representative run is shown for the inflow/outflow plots.

Supplementary Table S9. Average performance metrics for two PID configurations for test scenario 9

Scenario 9		
	Conservative	Aggressive
MDPE (%)	-4.86	-2.06
MDAPE (%)	4.86	2.09

**Steady-state overshoot (%)**	2.55	2.13
**Steady-state undershoot (%)**	-29.74	-29.91
**Target overshoot (%)**	0.05	0.61
**Effectiveness (%)**	74.35	88.70
**Wobble (%)**	1.11	0.90
**End-state divergence (%/hr)**	-11.00	1.54
**Rise time efficiency (min)**	3.53	2.36
**Volume efficiency**	2.34	2.36
**Median infusion rate (mL/min)**	44.31	57.43
**Mean infusion rate (mL/min)**	58.77	73.64
**Area above target pressure (min)**	0.00	0.02
**Area below target pressure (min)**	-2.03	-1.17
**Area to rise time (min)**	-0.93	-0.52
**Infusion rate variability (%)**	7.22	37.44

Supplementary Table S10. Average performance metrics for two PID configurations for test scenario 10

**Scenario 10**		
	**Conservative**	**Aggressive**
**MDPE (%)**	-10.75	-5.16
**MDAPE (%)**	10.75	5.16
**Steady-state overshoot (%)**	4.20	3.06
**Steady-state undershoot (%)**	-23.54	-27.61
**Target overshoot (%)**	0.00	0.00
**Effectiveness (%)**	14.22	90.95
**Wobble (%)**	0.70	0.53
**End-state divergence (%/hr)**	-3.38	-5.92
**Rise time efficiency (min)**	1.61	1.64
**Volume efficiency**	1.17	1.20
**Median infusion rate (mL/min)**	92.64	96.90
**Mean infusion rate (mL/min)**	92.40	105.97

Area above target pressure (min)	0.00	0.00
Area below target pressure (min)	-3.29	-1.77
Area to rise time (min)	-0.75	-0.45
Infusion rate variability (%)	6.94	35.03

Supplementary Table S11. Average performance metrics for two PID configurations for test scenario 11

Scenario 11		
	Conservative	Aggressive
MDPE (%)	-12.74	-6.36
MDAPE (%)	12.74	6.36
Steady-state overshoot (%)	1.83	1.70
Steady-state undershoot (%)	-20.88	-25.94
Target overshoot (%)	0.00	0.00
Effectiveness (%)	0.00	73.33
Wobble (%)	0.59	0.60
End-state divergence (%/hr)	-4.20	-1.93
Rise time efficiency (min)	1.47	1.97
Volume efficiency	1.14	1.22
Median infusion rate (mL/min)	110.48	137.75
Mean infusion rate (mL/min)	119.19	147.36
Area above target pressure (min)	0.00	0.00
Area below target pressure (min)	-4.21	-2.39
Area to rise time (min)	NA	-0.75
Infusion rate variability (%)	5.67	36.83

s

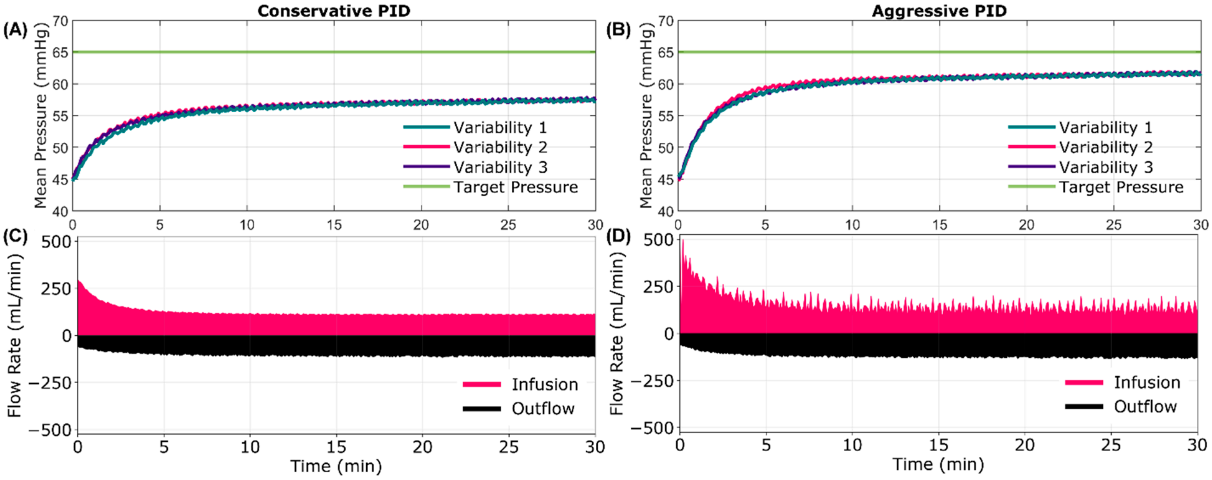

Supplementary Figure S7. Results for Scenario 11. Results for the Conservative (A, C) and Aggressive (B, D) tuned PID are shown for (A,B) MAP and (C,D) infusion and outflow rate as the controller tries to reach a target MAP of 65mmHg. For MAP plots, each of three subject variability runs are plotted individually while a single representative run is shown for the inflow/outflow plots.
